# Establishment and application of a high-throughput screening model for cell adhesion inhibitors

**DOI:** 10.3389/fphar.2023.1140163

**Published:** 2023-02-23

**Authors:** Han Sun, Xue-Kai Wang, Jian-Rui Li, Mei Tang, Hu Li, Lei Lei, Hong-Ying Li, Jing Jiang, Jia-Yu Li, Biao Dong, Jian-Dong Jiang, Zong-Gen Peng

**Affiliations:** ^1^ CAMS Key Laboratory of Antiviral Drug Research, Institute of Medicinal Biotechnology, Chinese Academy of Medical Sciences and Peking Union Medical College, Beijing, China; ^2^ Beijing Key Laboratory of Antimicrobial Agents, Institute of Medicinal Biotechnology, Chinese Academy of Medical Sciences and Peking Union Medical College, Beijing, China; ^3^ Key Laboratory of Biotechnology of Antibiotics, The National Health and Family Planning Commission (NHFPC), Institute of Medicinal Biotechnology, Chinese Academy of Medical Sciences and Peking Union Medical College, Beijing, China

**Keywords:** cell adhesion, high-throughput screening model, inhibitor, lifitegrast, acute liver injury

## Abstract

The cell adhesion between leukocytes and endothelial cells plays an important balanced role in the pathophysiological function, while excessive adhesion caused by etiological agents is associated with the occurrence and development of many acute and chronic diseases. Cell adhesion inhibitors have been shown to have a potential therapeutic effect on these diseases, therefore, efficient and specific inhibitors against cell adhesion are highly desirable. Here, using lipopolysaccharide-induced human umbilical vein endothelial cells (HUVECs) and calcein-AM-labeled human monocytic cell THP-1, we established a high-throughput screening model for cell adhesion inhibitors with excellent model evaluation parameters. Using the drug repurposing strategy, we screened out lifitegrast, a potent cell adhesion inhibitor, which inhibited cell adhesion between HUVEC and THP-1 cells by directly interrupting the adhesion interaction between HUVEC and THP-1 cells and showed a strong therapeutic effect on the mouse acute liver injury induced by poly (I:C)/D-GalN. Therefore, the screening model is suitable for screening and validating cell adhesion inhibitors, which will promote the research and development of inhibitors for the treatment of diseases caused by excessive cell adhesion.

## 1 Introduction

The cell adhesion between leukocytes and endothelial cells is a cascade process. Once activated by stimulating factors, such as lipopolysaccharide (LPS), tumor necrosis factor-alpha (TNF-α), and interleukin-1 beta (IL-1β), the endothelial cells upregulate the expression of cell-surface adhesion molecules, including selectins, integrins, immunoglobulin family molecules (vascular cell adhesion molecule-1 (VCAM-1) and intercellular adhesion molecule-1 (ICAM-1)) (Takahashi et al., 1996; Huang et al., 2018), and chemokines such as monocyte chemoattractant protein-1 (MCP-1) (Li et al., 2021), C-X-C motif ligand 10 (CXCL10) (Kawaguchi et al., 2021), and interleukin-8 (IL-8) (Chen et al., 2018). When rolling on the endothelial surface mediated with selectins, the leukocytes slow down and stagnate on the endothelial surface by the chemokines and adhesion molecules, and ultimately undergo migration across the endothelium and basement membrane through the interaction of integrins and their ligand, immunoglobulin family molecules on the surface of endothelial cells (Ley et al., 2007; Cook-Mills et al., 2011). Normally, cell adhesion plays an important balanced role in the pathophysiological function, while excessive adhesion results in many diseases, such as cardiovascular diseases (atherosclerosis, ischemia-reperfusion injury, thrombosis, and hypercholesterolemia) (Scalia et al., 1998; Blankenberg et al., 2003; Kawamura et al., 2004; McEver, 2015), chronic inflammatory diseases (chronic obstructive pulmonary disease, asthma (Woodside and Vanderslice, 2008), colitis (Sans et al., 1999), keratoconjunctivitis (Perez et al., 2016), and dermatitis (Jung et al., 1996)), autoimmune diseases (Giblin and Lemieux, 2006), and even the migration of tumor cells (Zimmerman and Blanco, 2008; Läubli and Borsig, 2010; Laferrière et al., 2004; O'Hanlon et al., 2002). Some adhesion inhibitors directly or indirectly interrupt the adhesive processes between leukocytes and endothelial cells and thus reduce the excessive cell adhesion, which contributes to the prevention and treatment of these acute and chronic diseases (Ulbrich et al., 2003; Perez et al., 2016). Up to date, only 18 adhesion inhibitors, with 12 monoclonal antibodies and 6 small molecule entities, have been approved by the US FDA for the treatment of related diseases (https://www.fda.gov/, 2022). Because of the low cost, easy oral administration, and good pharmacological effects, small molecule drugs are greatly attractive to research and development (Zimmerman and Blanco, 2008), and efficient and specific small molecule adhesion inhibitors are highly desirable to meet unresolved clinical needs.

To obtain small molecular adhesion inhibitors, a high-throughput screening model for cell adhesion inhibitors is needed to acquire innovative leading compounds. Currently, cell adhesion assays are mainly divided into static adhesion assay and the measurement of adhesion in shear stress (Kucik and Wu, 2005). Static adhesion assays are mainly used to assess the adhesion interaction between cells and extracellular matrix by measuring absorbance or fluorescence intensity (Tolosa and Shaw, 1996; Spessotto et al., 2002; Kucik and Wu, 2005; Humphries, 2009), while the cell adhesion in blood and lymph vessels is measured under shear stress using flow chambers and fluorescence microscopy (Kucik and Wu, 2005). A direct co-culture of two types of cells was established and detected with fluorescence microscopy (Li et al., 2007). For enhancing the adhesion intensity, endothelial cells were stimulated with inducible factors such as IL-1β (Zhong Y. et al., 2020), LPS (Jung et al., 1996; Wang et al., 2019; Zhong M. et al., 2020), TNF-α (Phang et al., 2020; Fallon and Hinds, 2021), or oxidized low-density lipoprotein (ox-LDL) (Gong et al., 2019; Geng et al., 2020). However, in those systems, the fluorescent signal from the labeled THP-1 captured by the activated endothelial cells was detected by fluorescence microscopy. These models are suitable to study the efficacy and mechanism of action of candidates, but not convenient for large-scale drug screening because of their time-consuming and labor-intensive.

In this study, we established a high-throughput screening model for cell adhesion inhibitors using LPS-induced human umbilical vein endothelial cells (HUVECs) and calcein-AM-labeled human monocytic cells THP-1, and lifitegrast, a drug that used for anti-dry eye therapy, was screened out from the FDA-approved drug library and shown to have a good therapeutic effect against mouse acute liver injury induced by poly (I:C)/D-GalN.

## 2 Materials and methods

### 2.1 Cell culture and reagents

HUVEC and THP-1 cells were from the Institute of Basic Medicine, Chinese Academy of Medical Sciences and Peking Union Medical College. HUVEC cells were incubated in Dulbecco’s modified eagle’s medium (DMEM, Gibco, China) supplemented with 10% (v/v) fetal bovine serum (FBS, Gibco, United States), 1% penicillin-streptomycin (Beyotime, Shanghai, China), 1% non-essential amino acids (Sigma-Aldrich, United States), and 0.01 mg/mL insulin (Psaitong, Beijing, China). THP-1 cells were maintained in Roswell Park Memorial Institute 1640 medium (RPMI 1640, Gibco, China) containing 10% (v/v) FBS, 1% penicillin-streptomycin, and 0.05 mM *β*-Mercaptoethanol (Sigma-Aldrich, United States). The cells were cultured at 37°C in a humidified incubator (Thermo, United States) with 5% CO_2_.

### 2.2 Cytotoxicity assay

The cytotoxicity was analyzed with a staining method. HUVEC cells (1 × 10^4^ cells/well) were seeded in a 96-well plate and treated with various concentrations of LPS for 24 h. Then, the culture supernatants were discarded, and the cells were incubated for 2 h with 10% CCK-8 (TransGen, Beijing, China) solution diluted with the culture medium. The absorbance intensity was measured at 450 nm using an Enspire Multilabel Reader (PerkinElmer, United States).

### 2.3 Establishment and optimization of the cell adhesion between HUVEC and THP-1 cells

HUVEC cells (1 × 10^4^ cells/well) were seeded in a 96-well plate pre-coated with type I collagen (Corning, United States). After 24 h of incubation, the cells were treated with 1 μg/mL of LPS for 24 h. THP-1 cells were labeled with 5 μM calcein-AM (Invitrogen, United States) for 30 min, then centrifuged at 200 g for 5 min, and resuspended with the culture medium of HUVECs prior to use. The labeled THP-1 cells (4 × 10^4^ cells/0.1 mL) were added to the well to co-culture with HUVEC cells for 45 min, then the cells were washed with phosphate buffer solution (PBS, Servicebio, China) using BioTek ELx50 Microplate Strip Washer (BioTek, USA) following the optimized procedure to remove the unadhered THP-1 cells. The fluorescence intensity was measured by Enspire Multilabel Reader (PerkinElmer, USA) with following parameters: top reading mode was used to measure fluorescence intensity at 490 nm excitation wavelength and 510 nm emission wavelength. A 5 × 5 rectangular array with 25 detection points was selected for each well with a distance of 0.72 mm between points to ensure maximum coverage of each well in the 96-well plate. The data for each well was output as the average of the fluorescence intensities of the 25 points.

### 2.4 The parameters of adhesion model and the screening of adhesion inhibitors

The key screening model parameters, signal-to-noise ratio (S/N), signal background ratio (S/B), coefficient of variation (CV), and Z factor (Z’), were calculated as follows (Zhao et al., 2019): S/N = (Mean_signal_—Mean_background_)/SD_background_, S/B = Mean_signal_/Mean_background_, CV = SD_control_/Mean_control_ × 100%, and Z’ = 1—(3SD_signal_ + 3SD_control_)/(Mean_signal_—Mean_control_). In screening, 2,791 compounds from the L1000-Approved Drug Library and L6000 Natural Product Library (Topscience Co. Ltd., Shanghai, China) were tested. HUVEC cells were incubated simultaneously with LPS and compound for 24 h, and then the labeled THP-1 cells were added to co-culture for 15 min. After the plate was washed, the fluorescence intensity (FI) was detected. The inhibitory activity of drugs against cell adhesion was calculated as follows: Inhibition rate (%) = (FI_drug_—FI_control_)/(FI_model_—FI_control_) × 100%.

### 2.5 Animal experiments

Six to eight-week-old male BALB/c mice (20.0 g ± 1.0 g) with SPF grade were from SPF (Beijing) Biotechnology Co., Ltd. All mice were housed under pathogen-free conditions with a standard 12h- light/dark cycle and fed sterile chow and fluid *ad libitum*. All animal procedures were performed strictly following the national laboratory animal feeding management standards and approved by the Institutional Animal Care and Use Committee of the Institute of Medicinal Biotechnology and Chinese Academy of Medical Sciences (SYXK (Jing)2017-0023).

The mice were randomly divided into 6 groups with 6 mice in each group according to body weight: normal control group, poly (I:C)/D-GalN model group, lifitegrast high-dose group (lifitegrast, 0.5 mg/kg), medium-dose group (lifitegrast, 0.25 mg/kg) and low-dose group (lifitegrast, 0.125 mg/kg), and dexamethasone group (dexamethasone, 1.0 mg/kg). Poly (I:C) (InvivoGen, thrl-picw-250, California) or D-(+)-galactosamine (D-GalN, Sigma-Aldrich, G1639-1G, United States) was diluted in 0.9% sterile saline. Dexamethasone (Dex, Innochem, Beijing, China) or lifitegrast (Aladdin, L171714, Shanghai, China) was diluted in 0.9% sterile saline containing 1% DMSO and 5% tween-80 (vehicle) prior to use.

The mice were administered intraperitoneally 500 mg/kg D-GalN in combination with 5 mg/kg poly (I:C) *via* the tail vein to induce acute liver injury, and the normal control group were treated with the equivalent volume of 0.9% sterile saline. The mice were treated intraperitoneally with the drugs at 2 and 10 h after the poly (I:C)/D-GalN injection, and the normal control group and poly (I:C)/D-GalN model group received the equivalent volume of vehicle. The blood samples were collected for biochemical assays, then the mice were sacrificed, and the liver tissues were collected for the following experiments after 18 h of the poly (I:C)/D-GalN injection.

### 2.6 Serum alanine aminotransferase measurement

The blood samples were centrifuged at 2,500 g for 10 min, and the sera were isolated. Serum levels of alanine aminotransferase (ALT) were detected with the ALT kits (Nanjing Jiancheng Bioengineering Institute, Nanjing, China) according to the manufacturer’s instructions.

### 2.7 Histopathological analyze

The histopathological changes in the liver were assessed after Hematoxylin and Eosin (H&E) staining. In brief, the liver tissue samples were flushed with PBS, fixed in 4% paraformaldehyde for 2 days, then dehydrated, and paraffin-embedded. The sections were performed H&E staining and scanned panoramically with Pannoramic Scan (3DHISTECH, Hungary).

### 2.8 Western blot

The liver tissues were homogenized and lysed in ice-cold lysis buffer supplemented with protease and phosphatase inhibitors (Topscience, Shanghai, China) and centrifuged at 12,000 g for 20 min at 4°C, and then the clarified supernatants were collected. Then proteins were quantified with the Pierce BCA Protein Assay Kit (Thermo, United States) assay according to the manufacturer’s instructions. The western blot was performed as previously described (Li et al., 2022a; Li et al., 2022b). Briefly, after SDS-PAGE and transmembrane, the target proteins were accordingly probed with first antibodies against GAPDH (10494-1-AP, 1:1,000, Proteintech), phospho-Stat3 (9145, 1:1,000, CST), Stat3 (9139, 1:1,000, CST), phospho-p38 MAPK (4511, 1:1,000, CST), p38 MAPK (8690, 1:1,000, CST), phospho-NF-κB p65 (3033, 1:1,000, CST), NF-κB p65 (6956, 1:1,000, CST), phospho-IκBα (2859, 1:1,000, CST), and IκBα (4814, 1:1,000, CST), respectively. After incubation with the corresponding HRP-conjugated secondary antibody, the signal of the target protein was detected using a ChemiDo XRS gel imager system (Bio-Rad, United States) with Immobilon Western Chemiluminescent HRP Substrate (Millipore, United States) and was scanned by ImageJ software. The ratio of the target protein was normalized to the internal control protein GAPDH, and fold-change was calculated relative to the control group.

### 2.9 Quantitative real-time reverse transcription polymerase chain reaction (qRT-PCR)

Total RNA was extracted from the liver tissues or HUVEC cells with Magen RNeasy Mini Kit (MGBio, Shanghai, China) according to the manufacturer’s instructions. RNA concentration was determined by NanoDrop 2000 (Thermo Scientific, United States). As previously described (Wang et al., 2022), qRT-PCR was performed with the indicated primers (Table 1) and HiScriptII One Step QRT-PCR SYBR Green Kit (Vazyme, Nanjing, China) using the ABI 7500 Fast system (Applied Biosystems, United States). RNA (30–120 ng) was used as a template, and qRT-PCR amplification consisted of 5 min of reverse transcription step at 50°C, then 5 min of an initial denaturation step at 95°C, followed by 40 cycles of PCR at 95°C for 10 s and 60°C for 34 s. Target gene expression levels were normalized to the internal control gene *GAPDH* using the 2^−ΔΔCT^ method.

**TABLE 1:** Primer sequences used in the qRT-PCR.

Gene name	Forward primer (5′-3′)	Reverse primer (5′-3′)
Human *GAPDH*	CGG​AGT​CAA​CGG​ATT​TGG​TCG​TAT	AGC​CTT​CTC​CAT​GGT​GGT​GAA​GAC
Human *ICAM-1*	TCT​TCC​TCG​GCC​TTC​CCA​TA	AGG​TAC​CAT​GGC​CCC​AAA​TG
Human *VCAM-1*	AAG​CCG​GAT​CAC​AGT​CAA​GTG	TCT​TGG​TTT​CCA​GGG​ACT​TC
Human *E-selectin*	CCG​AGC​GAG​GCT​ACA​TGA​AT	GCC​AGA​GGA​GAA​ATG​GTG​CT
Murine *GAPDH*	CTC​TGG​AAA​GCT​GTG​GCG​TGA​TG	ATG​CCA​GTG​AGC​TTC​CCG​TTC​AG
Murine *IL-1β*	TGT​CTT​GGC​CGA​GGA​CTA​AGG	TGG​GCT​GGA​CTG​TTT​CTA​ATG​C
Murine *IL-6*	CCA​TCC​AGT​TGC​CTT​CTT​GG	TGC​AAG​TGC​ATC​ATC​GTT​GT
Murine *TNF-α*	AGG​GTC​TGG​GCC​ATA​GAA​CT	CCA​CCA​CGC​TCT​TCT​GTC​TAC
Murine *ICAM-1*	GTGGCGGGAAAGTTCCTG	CGT​CTG​CAG​GTC​ATC​TTA​GGA​G
Murine *MCP-1*	TTA​AAA​ACC​TGG​ATC​GGA​ACC​AA	GCA​TTA​GCT​TCA​GAT​TTA​CGG​GT
Murine *CXCL10*	GAGCCTATCCTGCCCACG	GGAGCCCTTTTAGACCTT

### 2.10 Statistical analyses

All data were presented as mean ± standard error of the mean (SEM). The statistical significance of differences between two-group was analyzed by unpaired *t-tests* and multiple-group comparisons by one-way ANOVA using GraphPad Prism 8. The differences were considered significant at *p*-value <0.05.

## 3 Results

### 3.1 The optimization of the cell adhesion condition between endothelial and monocytic cells

**FIGURE 1:**
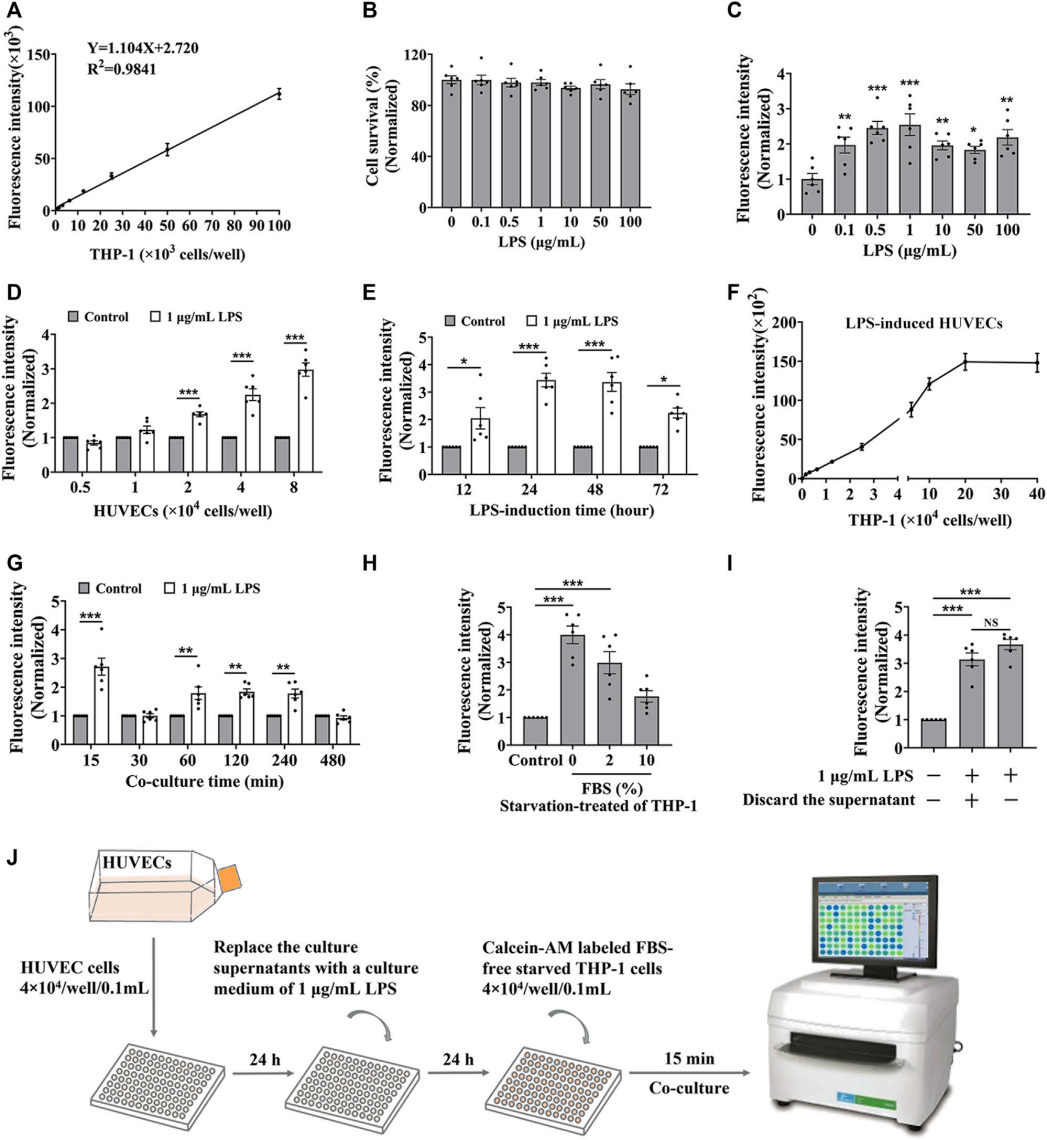
The optimization of the cell adhesion condition between endothelial and monocytic cells. **(A)** The linear relationship between fluorescence intensity and the amount of calcein-AM labelled THP-1 cells. **(B)** The cytotoxicity of LPS to HUVEC cells for 24 h. **(C–E)** HUVEC cells (4 × 10^4^ cells/well) were treated by LPS for 24 h **(C)**, or different densities of HUVEC cells were treated by 1 μg/mL LPS for 24 h **(D)**, or HUVEC cells (4 × 10^4^ cells/well) were treated by 1 μg/mL LPS for different time **(E)**, and then co-cultured with labeled THP-1 cells (4 × 10^4^ cells/well) for 45 min; or **(F–H)** HUVEC cells (4 × 10^4^ cells/well) were treated by 1 μg/mL LPS for 24 h, and then co-cultured with different amounts of labeled THP-1 cells for 45 min **(F)** or co-cultured with labeled THP-1 cells (4 × 10^4^ cells/well) for different time **(G)**, or co-cultured with labeled THP-1 cells (4 × 10^4^ cells/well) starved with media containing different concentrations of FBS for 15 min **(H)**, then the fluorescence intensity were detected. **(I)** The fluorescence intensity from the cell adhesion model when the culture supernatants from LPS induced-HUVEC cells were discarded before HUVEC cells (4 × 10^4^ cells/well) co-cultured with labeled THP-1 cells (4 × 10^4^ cells/well) for 15 min. **(J)** The technological diagram of the cell adhesion model between HUVEC and THP-1 cells. Data were presented as mean ± SEM over three experiments. NS: no significance, *p < 0.05, **p < 0.01, ***p < 0.001 vs. Control.

To establish the *in vitro* screening model for cell adhesion inhibitors, we used HUVEC endothelial cells and THP-1 monocytes to mimic the cell adhesion interaction (Zhong M. et al., 2020). Firstly, we evaluated whether the detectable fluorescence intensity is linear correlation with the amount of THP-1 cells labeled with calcein-AM. Results showed a good linear relationship between the fluorescence intensity and the amount of labeled THP-1 cells when it ranged from 0 up to 100 × 10^3^ cells/well in a 96-well plate (Figure 1A), indicating that the fluorescence intensity responds to the amount of the captured THP-1 cells. Normally, monocytes less adhere to endothelial cells, while the adhesion interaction was enhanced by LPS-stimulated endothelial cells (Lv et al., 2018). HUVEC cells did not show significant cytotoxicity by the treatment of LPS for 24 h (Figure 1B), while the adhesion of THP-1 cells to the HUVEC cells was enhanced by the LPS stimulation (Figure 1C). Considering the reaction curve and the potential cytotoxicity, we selected 1.0 μg/mL LPS as the stimulation concentration. At the stimulation of 1.0 μg/mL of LPS, THP-1 cells adhered to the stimulated HUVEC cells in an amount of HUVEC cells manner (Figure 1D). Considering the monolayer state of endothelial cells, HUVEC cells at the density of 4 × 10^4^ cells/well were treated by 1 μg/mL of LPS for different times, and results showed that the most vigorous adhesion appeared at 24 h of treatment (Figure 1E). Then we again verified whether there was a strong linear relationship between fluorescence intensity and the amount of THP-1 cells under 20 × 10^4^ cells/well when HUVEC cells were stimulated by LPS (Figure 1F). At this condition, the adhesion between the stimulated HUVEC cells and THP-1 cells was completed at 15 min without increasing fluorescence intensity by additional co-culture time (Figure 1G). However, the starvation state of THP-1 cells significantly influenced the adhesion, the strongest fluorescence intensity was observed when 0% FBS starved THP-1 cells were added (Figure 1H), while the culture supernatant from LPS induced-HUVEC cells did not influence the adhesion between the stimulated HUVEC cells and THP-1 cells (Figure 1I).

Together, we suggested that HUVEC cells, with a density of 4 × 10^4^ treated by 1 μg/mL of LPS for 24 h, were co-cultured with labeled FBS-free starved THP-1 cells at the density of 4 × 10^4^ cells/well for 15 min presented a good cell adhesion condition to mimic the cell adhesion interaction between HUVEC and THP-1 cells (Figure 1J).

### 3.2 The parameters of *in vitro* high-throughput screening model for cell adhesion inhibitors and the screening drug candidates

**FIGURE 2:**
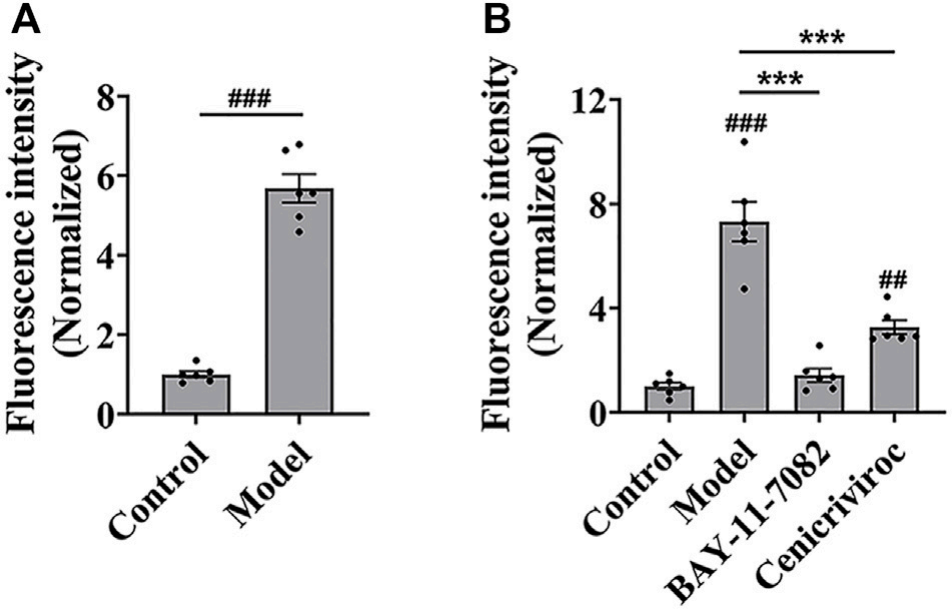
The evaluation of *in vitro* high-throughput screening model for cell adhesion inhibitors. **(A)** The reproducibility of the cell adhesion model between the stimulated HUVEC cells and labeled THP-1 cells. **(B)** The adhesion inhibitory activity of BAY-11-7082 and cenicriviroc (CVC) on cell adhesion. HUVEC cells were treated simultaneously by LPS and/or compound (BAY-11-7082 (10 μM) or CVC (1 μM)) for 24 h, and then co-cultured with labeled THP-1 cells for 15 min. Data were presented as mean ± SEM over three experiments. ^##^ *p* < 0.01, ^###^ *p* < 0.001 vs. control, and ***p < 0.001 vs. model.

Then, we evaluated whether the cell adhesion model is suitable for screening cell adhesion inhibitors. We analyzed the key evaluation parameters for the quality of high-throughput screening model, such as signal-to-noise ratio (S/N), signal background ratio (S/B), coefficient of variation (CV), and Z factor (Z’), among which Z’ is a measure of statistical effect intensity. Results showed that the cell adhesion interaction between the stimulated HUVEC cells and labeled THP-1 cells was reproducible (Figure 2A), with the S/N value of 10.3, S/B value of 9.8, CV value of 8.8%, and Z’ value of 0.7 (Table 2), strongly suggesting the cell adhesion model is suitable for the high-throughput screening of cell adhesion inhibitors. Activated endothelial tissue by LPS upregulate the expression of adhesion molecules and the secretion of chemokines through NF-κB signaling pathway to promote the adhesion interaction with leukocytes (Zhu et al., 2017; Dayang et al., 2019), while BAY-11-7082 as NF-κB inhibitor and cenicriviroc as chemokine receptor (CCR) 2/5 antagonist showed adhesion inhibitory activity *in vivo* and *in vitro* (D'Antoni et al., 2018; Wang et al., 2016). In our optimized model, they were also shown to be strong cell adhesion inhibitors, further suggesting the *in vitro* model is reliable for the screening of cell adhesion inhibitors (Figure 2B).

**TABLE 2:** The key evaluation parameters of *in vitro* high-throughput screening model.

Parameters	Value	Good range of values
Signal-to-noise ratio (S/N)	10.3	>3.0
Signal background ratio (S/B)	9.8	>3.0
Coefficient of variation (CV)	8.8%	<20.0%
Z factor (Z’)	0.7	0.5 ≤ Z’ < 1.0

Drug repurposing strategy is a good strategy for discovering new therapeutic uses of drugs. (Armando et al., 2020). Using this model, 2,791 compounds from the approved drug library and natural product library were tested, and 8 potential adhesion inhibitors were screened out (Table 3), which showed inhibitory activity against the cell adhesion between HUVEC and THP-1 cells at the drug concentration of 1.0 μM. Among which only lifitegrast, a drug for the treatment of dry eye (Chan and Prokopich, 2019), showed adhesion inhibition rate more than 50% and was comparable to that of cenicriviroc, which has the potential to be developed for the clinical treatment of other adhesion-related diseases.

**TABLE 3:** The effect on cell adhesion of approved drugs.

Drug (1.0 μM)	Chemical structure	Inhibition (%)
Cenicriviroc (CVC)	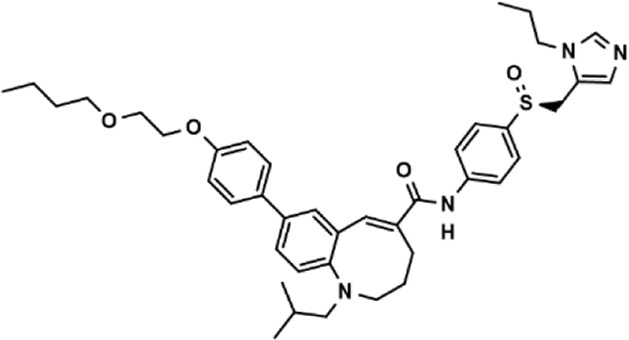	64.85 ± 3.76
Etomidate	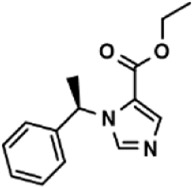	32.17 ± 8.55
Valacyclovir hydrochloride	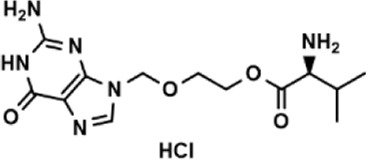	25.41 ± 1.53
Milrinone	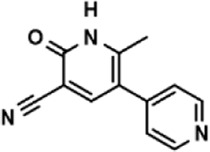	24.45 ± 7.94
Meclizine dihydrochloride	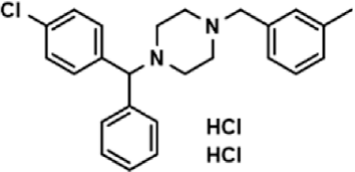	22.87 ± 3.01
Carprofen	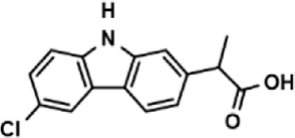	46.86 ± 19.4
Theobromine	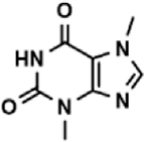	21.16 ± 12.54
Vorinostat	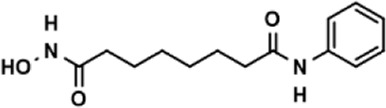	41.70 ± 5.39
Lifitegrast	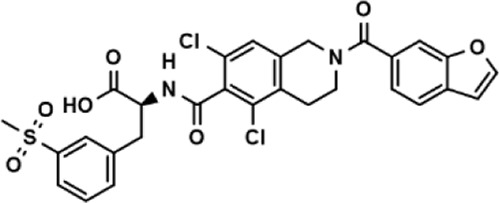	67.55 ± 22.99

### 3.3 Lifitegrast directly interrupts the cell adhesion between HUVEC and THP-1 cells

**FIGURE 3:**
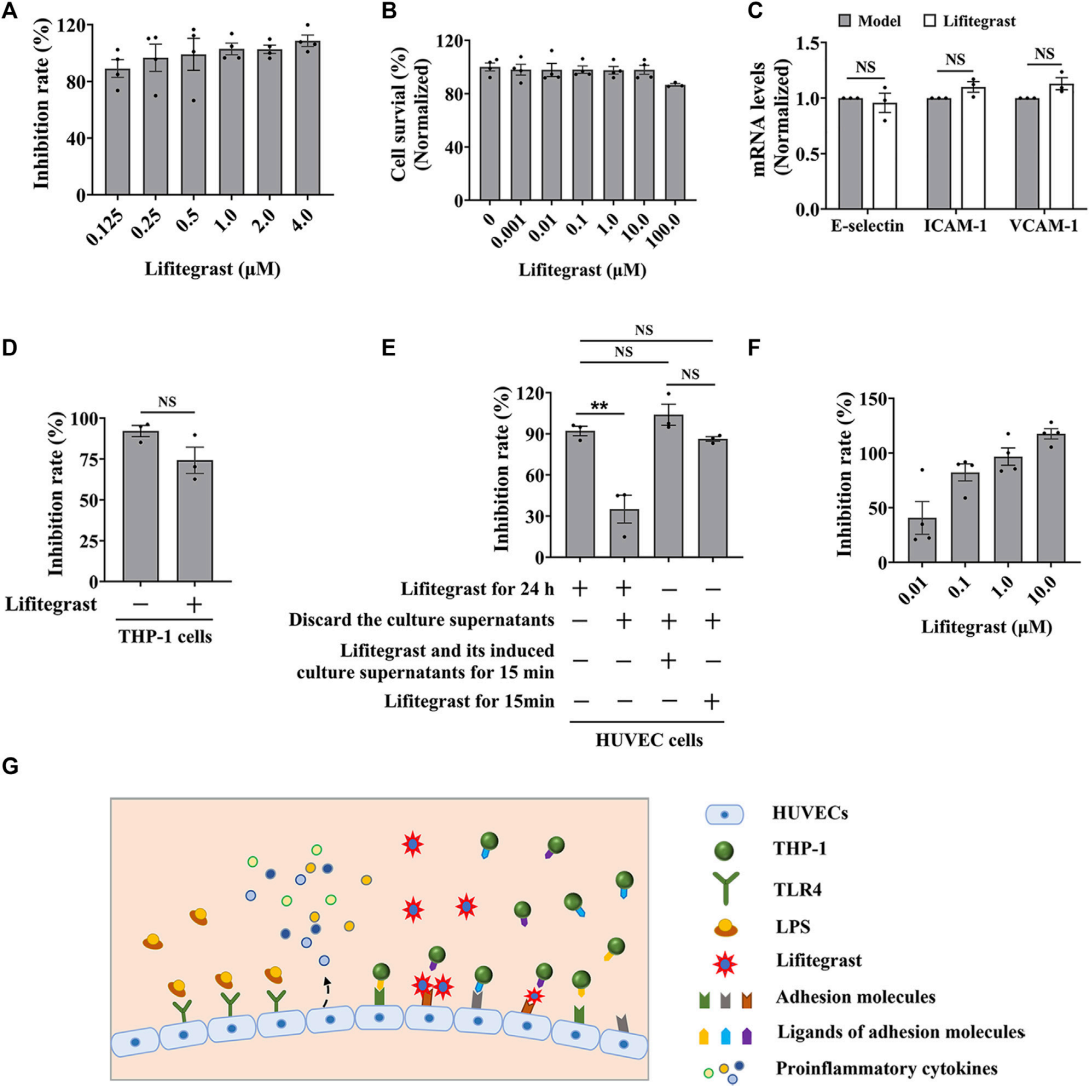
Lifitegrast directly interrupts the adhesion interaction between HUVEC and THP-1 cells. **(A)** The inhibitory activity of lifitegrast against cell adhesion in the standardized model. **(B)** The cytotoxicity of lifitegrast to HUVEC cells for 24 h. **(C)** The mRNA levels of adhesion molecules in HUVEC cells treated by LPS and lifitegrast for 24 h. **(D)** In the standardized model, the THP-1 cells were treated additionally with lifitegrast for 24 h. **(E)** HUVEC cells were treated at different stages of cell adhesion, such as adding lifitegrast together with LPS for 24 h, or discarding the culture supernatants of LPS and lifitegrast, or replacing the culture supernatants of LPS with that of lifitegrast, or with fresh culture medium containing lifitegrast, and then co-cultured with labeled THP-1 cells for 15 min. **(F)** HUVEC cells were treated by LPS for 24 h, the culture supernatants were replaced with culture media containing lifitegrast, and then co-cultured with labeled THP-1 cells for 15 min. **(G)** The diagram of lifitegrast against cell adhesion. Data were presented as mean ± SEM over three experiments. NS: no significance, **p < 0.01 vs. the group of retained culture supernatants.

We evaluated the efficacy of lifitegrast against cell adhesion using the standardized screening model. The results showed that lifitegrast inhibited the cell adhesion between HUVEC and THP-1 cells in a dose-dependent manner (Figure 3A) without significant cytotoxicity to HUVEC cells up to 10.0 μM of lifitegrast (Figure 3B). Then lifitegrast at 1.0 μM was used to explore the mechanism against cell adhesion between HUVEC and THP-1 cells.

Firstly, we detected whether lifitegrast influences the expression of adhesion molecules on the surface of HUVEC cells. Results showed that lifitegrast did not decrease the mRNA levels of LPS-induced ICAM-1, VCAM-1, and E-selectin (Figure 3C), which are the main adhesion molecules to promote cell adhesion between leukocytes and endothelial cells (Walzog and Gaehtgens, 2000; Huang et al., 2018; Zhong et al., 2018). Furthermore, additional treated THP-1 cells with lifitegrast for 24 h also did not enhance the inhibitory activity of lifitegrast against cell adhesion (Figure 3D). Those results suggested that the inhibitory activity of lifitegrast against cell adhesion is not through the impact of expressing the adhesion molecules on the cell surface.

Then we explored whether the cell culture supernatants of lifitegrast treatment will influence the cell adhesion between HUVEC and THP-1 cells. HUVEC cells were treated simultaneously by LPS and lifitegrast for 24 h, then the culture supernatants were discarded, and HUVEC cells were co-cultured with labeled THP-1 cells for 15 min. Results showed that the adhesion inhibitory activity of lifitegrast was decreased but not completely disappeared (Figure 3E), suggesting that some substances in the culture supernatants secreted from the treated HUVEC cells have an adhesion inhibitory effect, or lifitegrast directly impacts the cell adhesion.

We next distinguished the potential action of lifitegrast. HUVEC cells were treated by LPS for 24 h, then the culture supernatants were replaced with that of HUVEC cells treated by lifitegrast for 24 h, or with fresh culture medium containing lifitegrast, and the HUVEC cells were co-cultured with labeled THP-1 cells for 15 min. In the two conditions, the adhesion inhibitory activities of lifitegrast were as strong as that of HUVEC cells were treated simultaneously by LPS and lifitegrast for 24 h without replacing culture supernatants (Figure 3E), suggesting that lifitegrast may directly interfere with the cell adhesion interaction. Meanwhile, the adhesion inhibitory activity of lifitegrast increased in a dose-dependent manner with a half maximal inhibitory concentration (IC_50_) of 25.00 ± 25.28 nM, when the culture supernatants were replaced with fresh culture media containing various concentrations of lifitegrast (Figure 3F), further validating the direct inhibitory activity of lifitegrast against cell adhesion between HUVEC and THP-1 cells. Our results are consistent with previous reports that lifitegrast blocks the interaction of lymphocyte function-associated molecule (LFA-1) with its cognate ligand ICAM-1 by binding to LFA-1 (Perez et al., 2016; Chan and Prokopich, 2019).

Collectively, our results suggested that lifitegrast may directly interrupt the interactions between adhesion molecules on the surface of HUVEC and THP-1 cells and thus inhibit cell adhesion (Figure 3G).

### 3.4 Lifitegrast ameliorates mouse acute liver injury induced by poly (I:C)/D-GalN

**FIGURE 4:**
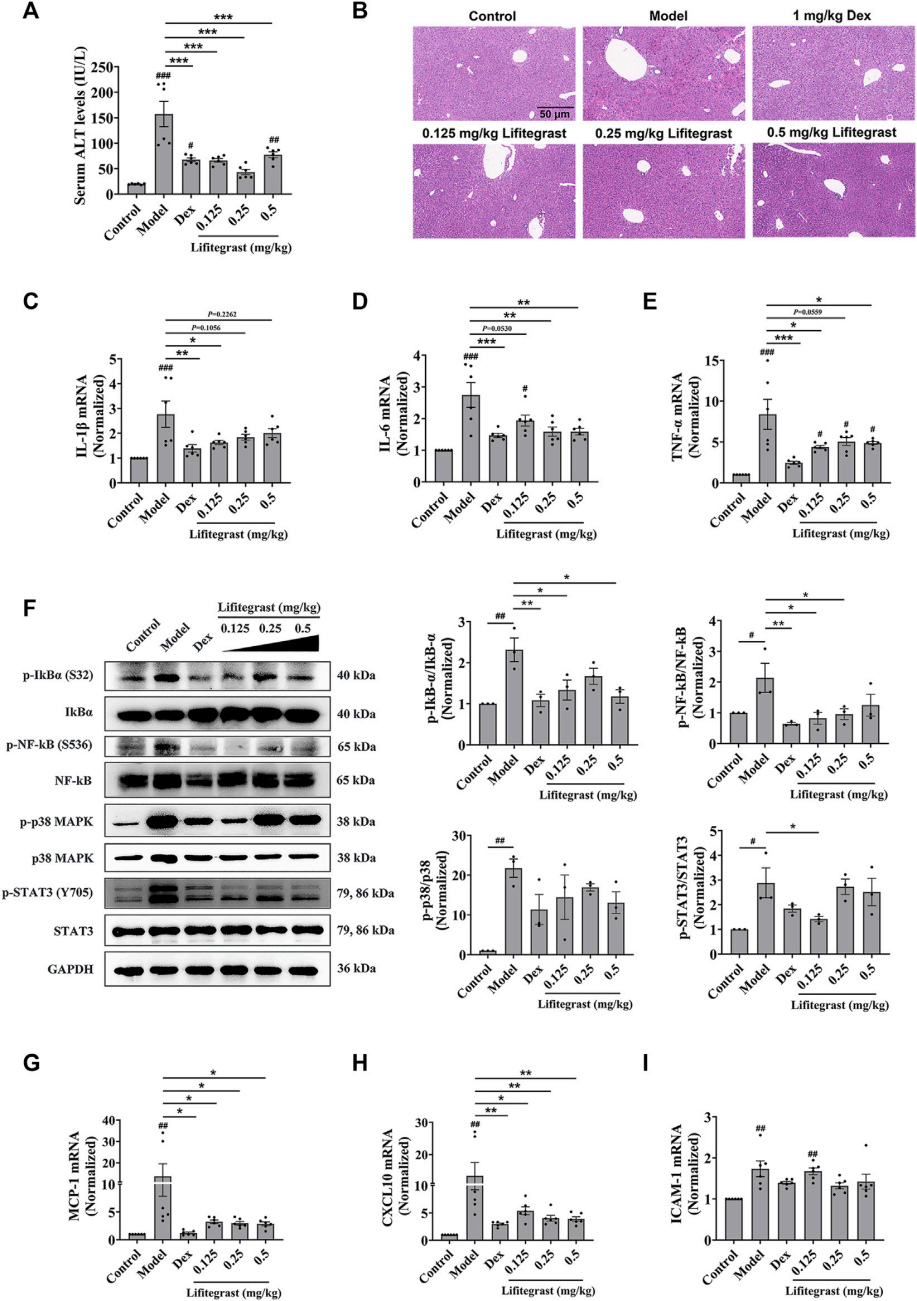
Lifitegrast ameliorates mouse acute liver injury induced by Poly(I:C)/D-GalN. **(A)** ALT level in serum, *n* = 6. **(B)** Liver H&E staining, the scale bar was 50 µm. **(C–E)** The mRNA levels of IL-1β **(C)**, IL-6 **(D)**, and TNF-α **(E)** in the liver, *n* = 6. **(F)** The phosphorylation levels of inflammatory factors in the liver, *n* = 3. **(G–I)** The mRNA levels of MCP-1 **(G)**, CXCL10 **(H)**, and ICAM-1 **(I)** in the liver, *n* = 6. Data were presented as mean ± SEM. ^#^ *p* < 0.05, ^##^ *p* < 0.01, ^###^ *p* < 0.001 vs. Control, and *p < 0.05, **p < 0.01, ***p < 0.001 vs. Model.

To further investigate the effect on cell adhesion of lifitegrast *in vivo*, we used a mouse acute liver injury model induced by poly (I:C)/D-GalN, which is closely related to the excessive cell adhesion condition (An et al., 2017). Here, we used male mice, which are mostly selected in the similar experiments (Gong et al., 2019; Lv et al., 2020; Schneider et al., 2021), to mimic acute liver injury. After the mice were intraperitoneally injected with poly (I:C)/D-GalN, the serum ALT level was significantly increased (Figure 4A), and H&E staining results showed increased infiltration of inflammatory cells in the liver tissue (Figure 4B). Consequently, inflammatory factors in the liver, such as IL-1β (Figure 4C), IL-6 (Figure 4D), and TNF-α (Figure 4E) were increased in parallel, suggesting that the treatment of poly (I:C)/D-GalN caused mouse acute liver injury. While the intraperitoneal injection treatment of lifitegrast ameliorated the acute liver injury by decreasing the ALT level (Figure 4A) and reducing the expression of inflammatory factors (Figures 4C–E), and the efficacy of lifitegrast was comparable to that of dexamethasone (Dex) (Figures 4A–E).

Then we validated the potential mechanism of cell adhesion of lifitegrast against the poly (I:C)/D-GalN-induced acute liver injury in mice. After the mice were treated with poly (I:C)/D-GalN, the phosphorylation levels of Stat3, p38 MAPK, IκBα, and NF-κB were increased in the liver (Figure 4F). The chemokines such as MCP-1 (Figure 4G) and CXCL10 (Figure 4H), and adhesion molecule ICAM-1 (Figure 4I) were increased in parallel, suggesting that poly (I:C)/D-GalN induced the excessive cell adhesion and caused acute liver injury. While the intraperitoneal injection treatment of lifitegrast ameliorated the acute liver injury by decreasing the phosphorylation levels of Stat3, p38 MAPK, IκBα and NF-κB (Figure 4F) and reducing the expression of chemokines (Figures 4G, H), but not ICAM-1 (Figure 4I), and the efficacy of lifitegrast was comparable to that of Dex (Figures 4F–I). These results suggested that lifitegrast ameliorates mouse acute liver injury through down-regulating the excessive cell adhesion.

## 4 Discussion

The occurrence and development of many acute and chronic diseases are closely related to excessive cell adhesion. In response to stimulating factors, vascular endothelial cells release pro-inflammatory cytokines and upregulate the expression of adhesion molecules on the cellular surface, thereby promoting intravascular inflammatory cells to adhere to the endothelium and undergo trans-endothelium migration (Huang et al., 2018). Using LPS-induced HUVEC cells and calcein-AM-labeled THP-1 cells, we established a screening model for cell adhesion inhibitors, achieving rapid, objective and high-throughput screening for cell adhesion inhibitors. HUVEC cells are used as typical endothelial cells for cell adhesion model (Cao et al., 2017). LPS, as an endotoxin, promotes the expression of pro-inflammatory cytokines and adhesion molecules on the cellular surface by acting on toll-like receptor 4 in endothelial cells and activating inflammation-related signaling pathways such as NF-κB signaling pathway (Su et al., 2021). In this study, LPS significantly induced excessive cell adhesion between HUVEC and THP-1 cells, which is consistent with previous reports (Hayden and Ghosh, 2008; Sawa et al., 2008; Lv et al., 2016). However, due to the static mode, this model is not suitable for the screening inhibitors for chemotactic factors which also play important roles in the excessive cell adhesion *in vivo* (Kim, 2004; Maas et al., 2023).

Drug repurposing strategy is a good strategy for discovering new therapeutic uses of drugs. Using the screening model, we obtained a potential cell adhesion inhibitor lifitegrast. And using the *in vitro* adhesion model, we verified that lifitegrast directly inhibited the cell adhesion between HUVEC and THP-1 cells, which is consistent with that lifitegrast blocks the interaction of LFA-1 with its cognate ligand ICAM-1 by binding to LFA-1 (Perez et al., 2016; Chan and Prokopich, 2019), further suggesting that the screening model is practicability. Although approved as a novel integrin antagonist, lifitegrast is used only for the treatment of the dry eye disease. In this study, we demonstrated that lifitegrast also shows a new potential therapeutic usage to treat acute liver injury. The detailed mechanism was associated with not only direct interruption of cell adhesion between leukocytes and endothelial cells but also downregulation of activated inflammatory signaling pathways.

In addition, acute liver failure (ALF) is a rare and serious consequence of acute fulminate liver cell damage, which can develop into a fatal outcome within days or weeks in the clinic (Bernal et al., 2010; Thawley, 2017; Stravitz and Lee, 2019). In terms of etiology, viral hepatitis may account for the majority of ALF cases in developing countries (Dong et al., 2020). In addition, as of May 20, 2022 there have been at least 566 probable cases of acute hepatitis of unknown cause in children under the age of 10 reported from 33 countries (WHO, 2022). Although the cause is unknown, given the epidemiological pattern of cases, it is presumed that these cases are associated with viral infection and may develop to acute liver failure in children (Christie, 2022). Many studies have shown that the infiltration of inflammatory cells in the liver is the key to the mechanism and result of ALF. Under the stimulation of inflammation, the expressions of ICAM-1 and VCAM-1 on the surface of liver sinusoidal endothelial cells (LSECs) are increased and chemokines are secreted, which mediates the adhesion cascade of inflammatory cells and the infiltration in the liver (Lalor and Adams, 2002; Bernal et al., 2010; Sørensen et al., 2015). Then inflammatory cells further cause hepatocyte damage through the direct activation of death receptors or the secretion of cytokines until liver failure (Jaeschke, 2006; Jaeschke and Hasegawa, 2006; Quaglia et al., 2008). Therefore, reasonable intervention for the adhesion process can block liver injury at an early stage to improve ALF. The polyinosine polycytidylic acid (poly (I:C)), a synthetic mimic of double-stranded viral RNA, is commonly used to mimic a moderate acute hepatic injury and a model of viral hepatitis (Takeda and Akira, 2004; Hafner et al., 2013; He et al., 2013; Khan et al., 2021). In this study, the intraperitoneal injection of D-GalN combined with tail vein injection of poly (I:C) caused acute liver injury in mice, and lifitegrast, a drug screened out with the *in vitro* screening model, ameliorated the acute liver injury, suggesting that lifitegrast might be a candidate for the treatment of acute liver failure though it needs to be validated further.

In summary, using LPS-induced HUVEC cells and calcein-AM-labeled THP-1 cells, we established a high-throughput screening model for cell adhesion inhibitors. Using the drug repurposing strategy, we screened out lifitegrast as a potent cell adhesion inhibitor with directly interrupting cell adhesion interaction between HUVEC and THP-1 cells, and further demonstrated the therapeutic effect on the mouse acute liver injury induced by poly (I:C)/D-GalN. Together, the screening model is suitable for screening cell adhesion inhibitors and validating their mechanism of action, which will promote the research and development of small molecular candidates against cell adhesion.

## Data Availability

The original contributions presented in the study are included in the article/supplementary material, further inquiries can be directed to the corresponding author.
